# Prevalence and patterns of vitamin D deficiency and its role in cognitive functioning in a cohort from South India

**DOI:** 10.1038/s41598-024-62010-5

**Published:** 2024-05-16

**Authors:** Aishwarya Ghosh, Monisha S, Albert Stezin Sunny, Latha Diwakar, Thomas Gregor Issac

**Affiliations:** Centre for Brain Research, Indian Institute of Science Campus, CV Raman Avenue, Bangalore, 560012 India

**Keywords:** Vitamin D, Deficiency, Prevalence, Cognitive functioning, Verbal fluency, Dyslipidemia, Biochemistry, Neuroscience, Risk factors

## Abstract

Vitamin D (VitD) is a naturally occurring, fat-soluble vitamin which regulates calcium and phosphate homeostasis in the human body and is also known to have a neuroprotective role. VitD deficiency has often been associated with impaired cognition and a higher risk of dementia. In this study, we aimed to explore the relationship between levels of VitD and cognitive functioning in adult individuals. 982 cognitively healthy adults (≥ 45 years) were recruited as part of the CBR-Tata Longitudinal Study for Aging (TLSA). Addenbrooke’s cognitive examination-III (ACE-III) and Hindi mental status examination (HMSE) were used to measure cognitive functioning. 25-hydroxyvitamin D [25(OH)D] levels were measured from the collected serum sample and classified into three groups— deficient (< 20 ng/ml), insufficient (20–29 ng/ml) and normal (≥ 30 ng/ml). Statistical analysis was done using IBM SPSS software, version 28.0.1.1(15). The mean age of the participants was 61.24 ± 9 years. Among 982 participants, 572 (58%) were deficient, 224 (23%) insufficient and only 186 (19%) had normal levels of VitD. Kruskal–Wallis H test revealed a significant difference in age (*p* = 0.015) and education (*p* = 0.021) across VitD levels and the Chi-square test revealed a significant association between gender (*p* = 0.001) and dyslipidemia status (*p* = 0.045) with VitD levels. After adjusting for age, education, gender and dyslipidemia status, GLM revealed that individuals with deficient (*p* = 0.038) levels of VitD had lower scores in ACE-III verbal fluency as compared to normal. Additionally, we also found that 91.2% individuals who had VitD deficiency were also having dyslipidemia. It is concerning that VitD deficiency impacts lipid metabolism. Lower levels of VitD also negatively impacts verbal fluency in adult individuals. Verbal fluency involves higher order cognitive functions and this result provides us with a scope to further investigate the different domains of cognition in relation to VitD deficiency and other associated disorders.

Vitamin D (VitD) is a naturally occurring fat-soluble vitamin that is endogenously synthesized in our body when the skin is exposed to sunlight^1^. Besides its role in calcium and phosphate metabolism, VitD has neurotropic roles and promotes nerve growth factors which have implications for brain health^1,2^.

The growing body of research suggests that low serum VitD levels are associated with impaired cognition with a high risk of dementia and Alzheimer’s Disease (AD)^2,3^. A meta-analysis by Goodwill and Szoeke, found that in both cross-sectional and longitudinal studies, lower levels of VitD were associated with cognitive decline^4^. Vedak et al. found that individuals with Mild Cognitive Impairment (MCI) and dementia exhibited low serum VitD levels and high homocysteine levels, indicating low VitD to be a marker of cognitive decline^5^. Low levels of VitD are also associated with a decline in executive functioning and episodic memory which are the two major domains affected in dementia and AD^6,7^.

Cognitive impairment is a major issue in the aging population and although a significant number of studies suggest the importance of VitD in cognition, the results are mixed ^8–10^. Yang et al. suggested that VitD supplementation improves the cognitive functioning of individuals suffering from MCI^9^. Other clinical studies found no improvement in cognition with VitD supplementation in individuals with MCI or mild to moderate AD^11,12^. Results from the randomized control trial study on Vitamin D, Omega-3 Fatty Acids and Cognitive Decline (VITAL-Cog), suggested VitD3 supplementation to have no significant impact on cognitive functioning in older adults^13^.

The endogenous production of VitD is greatly dependent on the geographical region in which an individual resides. It is assumed that individuals residing in regions away from direct exposure to sunlight will in general have a deficiency of VitD. This idea led to several studies on VitD in the Western populations but are limited in number in tropical countries like India^14^. In this scenario, it is important to explore the patterns of VitD levels and their effect on the cognitive functioning of individuals residing in a region that is well exposed to sunlight throughout the year.

The optimal level of VitD requirement for the human body has always been a debatable topic and the recommendations between several expert advisory bodies vary. The adequate level of VitD as per the Institute of Medicine is 20 ng/ml ^15^. In contrast, the US Endocrine Society and the International Osteoporosis Foundation define VitD levels as—“Deficient” (< 20 ng/ml), “Insufficient” (20–29 ng/ml) and “Sufficient” (> 30 ng/ml)^16,17^.

These insights led us to explore the patterns of VitD and its role in cognitive functioning of aging individuals from southern India. Our hypothesis posits that individuals with low levels of VitD will more likely exhibit impaired cognitive functioning.

## Materials and methods

### Study population

The subjects for this study consisted of 982 (494 males and 488 females), adult (≥ 45 years) individuals who are part of the Tata Longitudinal Study for Aging (TLSA)—a longitudinal observational study investigating cognitive trajectories of adult individuals. The cohort consist of participants from urban Bangalore, the capital city of Karnataka State in the southern part of India, wherein we recruit adults (middle aged and above) without dementia and perform detailed clinical, cognitive, imaging and genetic assessments to understand their risk and protective factors predisposing them to dementia and other neurodegenerative disorders. This is along the lines with Longitudinal Ageing Study in India (LASI) which is the first longitudinal study on aging in India, Canadian Longitudinal Study on Aging (CLSA) and Chinese Health and Retirement Longitudinal Study (CHARLS)^18–20^.

The prevalence of dementia due to modifiable risk factors (hearing loss, traumatic brain injury, hypertension, alcohol use and obesity) cumulatively come up to 15% in midlife as reported by the Lancet Commission^21^. Also, there are associated cognitive performance measures getting affected with perimenopausal age and less studied in our population^22,23^. Due to these factors, it is beneficial to look at cognitive trajectories in adults (middle-aged and above) without dementia (Clinical Dementia Rating Score < 1) and follow them up regularly to identify pre-dementia syndromes (Subjective Cognitive Decline, Motoric Cognitive Risk Syndrome, Mild Cognitive Impairment) while comprehensively evaluating for risk factors and comorbidities (diabetes, features of metabolic syndrome like hypertension, dyslipidemia etc.) and protective factors (multilingualism, musical training, physical activity) ^21,24–26^.

All our participants (*n* = 982) had normal cognitive functioning as indicated by Clinical Dementia Rating (CDR) score of zero. The CDR is a well-known global standard measure of dementia with high reliability and validity^27^. The TLSA Study protocol follows all ethical guidelines ensuring participants’ safety, informed consent, confidentiality of data and voluntary participation, and has been approved by the Institutional Ethics Committee of Centre for Brain Research, IISc. Informed consent was obtained from all the participants prior to the study.

### Serum 25(OH)D measurement

Blood samples were collected for all the participants of the TLSA and analysed using chemiluminescence immunoassays on VITROS ECiQ Immunodiagnostic System using Intellicheck® Technology. VitD status was determined by levels of metabolite 25(OH)D which is the most reliable clinical indicator^28^ and was categorised into three groups as per the guidelines of the Endocrinology Society of America—Deficient (< 20 ng/ml), Insufficient (20–29 ng/ml) and Normal (≥ 30 ng/ml)^16^.

### Cognitive Assessments

For this study cognitive functioning of the participants were assessed using two tools:

### Addenbrooke’s cognitive examination-III (ACE-III)

It is a cognitive assessment tool with high sensitivity to assess both global and domain specific cognitive functioning. It has high efficacy in diagnosis of MCI. This tool has tests for memory, attention, orientation, language, visuo-perceptual and visuo-spatial abilities. The maximum score of 100 is distributed across the different domains ^29^. The ACE-III has been translated to multiple Indian languages like Hindi, Tamil, Telegu, Kannada, Malayalam, Gujarati, Urdu and Indian English due to the socio-cultural and lexical relevance and has been validated^29–31^.

### Hindi mental status examination (HMSE)

It is the Indian adaptation of the Mini Mental Status Examination (MMSE) consisting of time bound subtests and is a popular measure of global cognition. The HMSE was developed as part of the Indo-US Cross-National Dementia Epidemiology Study that compared cognitive functioning between two rural populations of the US and India. A culturally and lexically relevant and sensitive version each item of the MMSE was modified according to the need of the Indian population and validated^32^.

### Statistical Analysis

Statistical Package for Social Sciences (SPSS) version 28.0.1.1(15) was used for all statistical analysis. Kolmogorov–Smirnov test of normality was used to check the distribution of the variables in the dataset. To find the mean differences between the demographic features and VitD levels, Kruskal–Wallis H test was used. The association between the categorical variables were established using chi-square test of significance. Generalized Linear Regression Model (GLM) was performed to find the relationship between cognitive scores across VitD levels. Significant variables (age, education, gender, and dyslipidemia status) from Kruskal Wallis and chi-square tests were considered as covariates and added to the regression model. Statistical significance was considered at *p* < 0.05.

## Results

### Demographic characteristics

**Figure 1:**
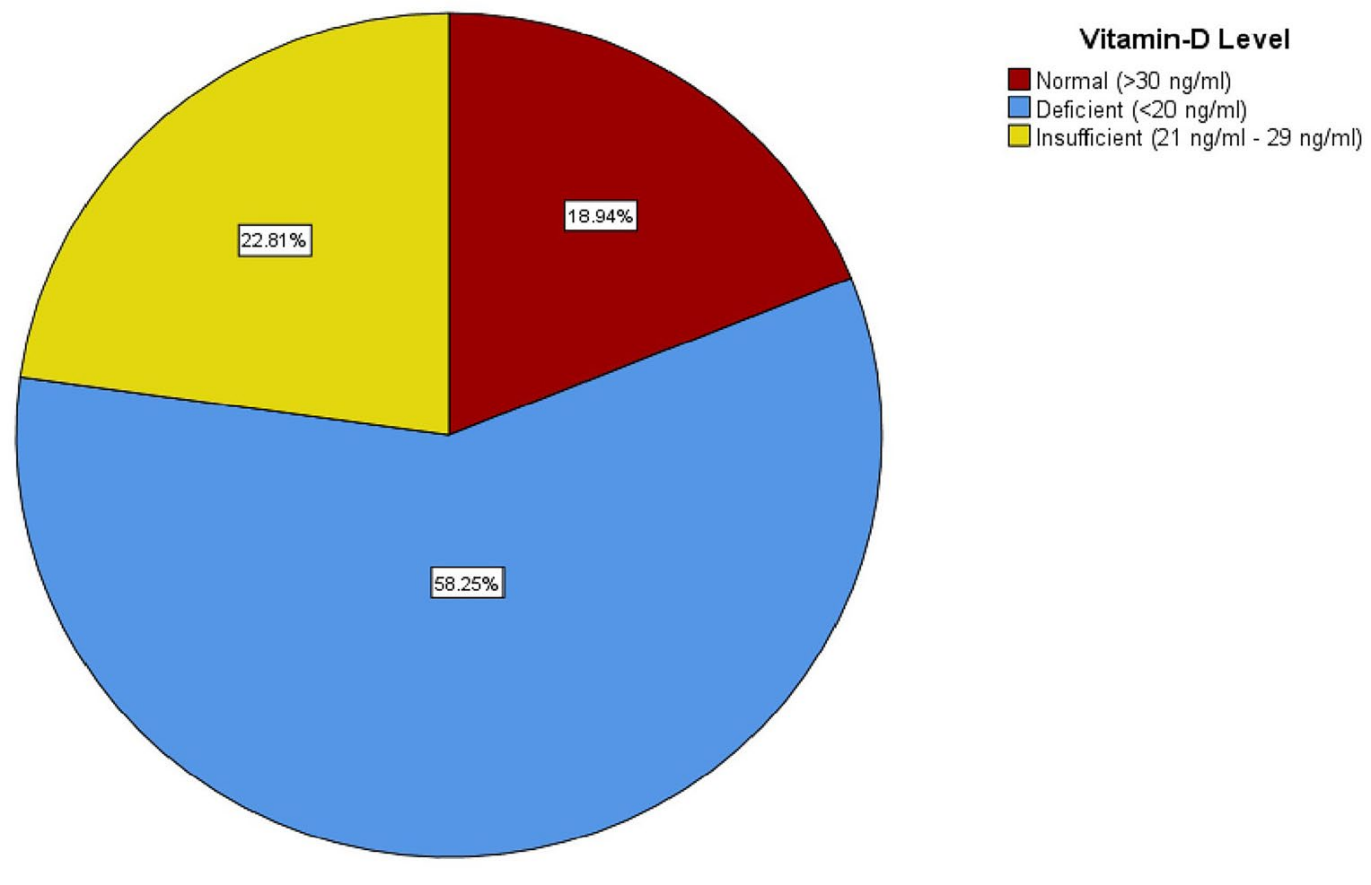
Prevalence of Vitamin D Deficiency. [Red square] Normal (> 30 ng/ml). [Blue square] Deficient (< 20 ng/ml). [Yellow square] Insufficient (21–29 ng/ml).

Out of the 1092 participants who had Vitamin D levels at the baseline, 110 participants had a CDR score above zero which indicated impaired cognitive functioning and were thus excluded from analysis. 982 participants who had a CDR score of zero, indicative of normal cognitive functioning, were included in this study. The mean age of the participants was 61.24 ± 9.00 years. Our cohort consisted of 494 males and 488 females. The mean VitD level of the cohort was 21.54 ± 12.60 ng/ml. There was a gross deficiency of VitD in our cohort with a total of 572 (58%) participants with VitD levels below 20 ng/ml, 224 (23%) had insufficient levels (20-29 ng/ml) and only 186 (19%) of individuals had normal levels (≥ 30 ng/ml) of VitD (Fig. 1).

Kolmogorov–Smirnov was performed to test for the distribution of variables, which were found to be not normally distributed.

The demographic details of the study population are summarized in Tables 1 and 2. Kruskal–Wallis H test revealed a significant difference in age (*p* = 0.015) and education (*p* = 0.021) across VitD levels. Chi-square test revealed a significant association with gender (*p* = 0.001) and dyslipidemia status (*p* = 0.045) and the three levels of VitD. There was no significant difference between the included participants with respect to socioeconomic status and dietary patterns.

**Table 1:** Mean ± SDs of demographic features of the study sample.

Demographic characteristics	
25(OH)D (ng/ml)	21.54 ± 12.60
Age (in years)	61.24 ± 9.00
Education (in years)	14.56 ± 4.33
Gender (percentage)	
Male	494 (50.3%)
Female	488 (49.7%)

*ng/ml* nanogram per millilitre.

**Table 2:** Mean ± SDs of demographic features of the study sample across levels of vitamin D.

Demographic Characteristics	Levels of 25(OH)D (ng/ml)			*p-*value
	Normal (≥ 30) [n = 186]	Insufficient (20–29) [n = 224]	Deficient (< 20) [n = 572]	
Age (in years)	61.75 ± 9.11	62.34 ± 8.90	60.64 ± 8.97	0.015*
Education (in years)	15.14 ± 4.08	14.26 ± 4.89	14.51 ± 4.16	0.021*
Gender (percentage)				
Male	39.10%	47.30%	54.90%	0.001*
Female	60.90%	52.70%	45.10%	
Dyslipidemia status (percentage)				
Yes	15.20%	10.30%	8.80%	0.045*
No	84.80%	89.70%	91.20%	

*ng/ml* nanogram per millilitre.

(*) indicates significant at p < 0.05.

Kruskal–Wallis H test have been used for age and education (continuous variables) and chi-square test have been used for gender and dyslipidemia status (categorical variables).

Additionally, 91.2% of individuals who had VitD deficiency in our cohort, were also found to have dyslipidemia. Since a high prevalence of dyslipidemia was found, we performed further analysis to check for an association between different lipid parameters and VitD levels. The cut-off for dyslipidemia was considered according to the National Cholesterol Education Program—Adult Treatment Pannel III (NCEP─ATP III) criteria [triglycerides ≥ 150 mg/dl or high-density lipoprotein (HDL) < 50 mg/dl or low-density lipoprotein (LDL) > 100 mg/dl]^33,34^. Chi-square test revealed that there existed a significant association of triglyceride (*p* = 0.021), HDL (*p* = 0.002) as well as LDL (*p* = 0.047) levels with VitD deficiency. Individuals with VitD deficiency had abnormal levels of the three lipid parameters as compared to normal (Table. 3).

**Table 3:** Percentage of individuals with abnormal lipid levels across the three levels of vitamin D.

Lipid parameters	Levels of 25(OH)D (ng/ml)		
	Normal (≥ 30) (%)	Insufficient (20–29) (%)	Deficient (< 20) (%)
Triglycerides			
< 150mg/dl	21.30	23.80	54.90
≥ 150 mg/dl	15.40	21.40	63.30
HDL			
≥ 50 mg/dl	24.90	23.90	51.10
< 50 mg/dl	16.30	22.30	61.40
LDL			
≤ 100mg/dl	23.20	23.55	53.40
> 100mg/dl	17.00	22.50	60.40

*ng/ml* nanogram per millilitre; *mg/dl* milligram per decilitre; *HDL* High-density lipoprotein; *LDL* Low-density lipoprotein.

### Neurocognitive findings

The findings of the neurocognitive measures have been summarized in Table 4. Kruskal–Wallis H test revealed a significant difference in ACE-III Attention (*p* = 0.016) and Fluency (*p* = 0.004) scores across the different levels of VitD.

**Table 4:** Mean ± SDs of neurocognitive measures across levels of vitamin D.

Neurocognitive measures	Levels of 25(OH)D (ng/ml)			*p-value*
	Normal (≥ 30)	Insufficient (20–29)	Deficient (< 20)	
ACE-III (Total)	92.23 ± 7.03	91.00 ± 7.27	91.93 ± 6.02	0.116
Attention	16.95 ± 1.37	16.67 ± 1.52	16.97 ± 1.37	0.016*
Memory	23.69 ± 2.98	23.57 ± 2.60	23.79 ± 2.43	0.539
Fluency	12.11 ± 2.01	11.60 ± 2.20	11.63 ± 1.96	0.004*
Language	24.81 ± 1.75	24.76 ± 1.89	24.90 ± 1.67	0.642
Visuospatial	14.67 ± 1.74	14.40 ± 1.91	14.64 ± 1.63	0.201
HMSE	30.40 ± 0.80	30.37 ± 0.92	30.41 ± 0.88	0.873

*ACE-III* Addenbrooke’s cognitive examination-III; *HMSE* Hindi mental status examination

(*) indicates significant at *p* < 0.05.

Kruskal Wallis-H test revealed a significant difference across the levels of 25(OH)D in ACE-III Attention and Fluency domain (not adjusted for age and gender).

GLM (unadjusted) revealed that individuals with insufficient VitD levels had significantly poorer scores in attention tasks as compared to that of normal levels of VitD (*p* = 0.047). Individuals with insufficient (*p* = 0.015) and deficient (*p* = 0.007) VitD levels also scored lesser in fluency domain of ACE-III. Even after adjusting for age, education, gender and dyslipidemia status, GLM revealed significantly lower verbal fluency scores in individuals with deficient (*p* = 0.038) levels of VitD as compared to that of normal (Table 5).

**Table 5:** Generalized Linear Regression Models.

Demographic features	Model 1 (Unadjusted) (95% C.I.)	*p*- value	*df*	Model 2 (Adjusted) (95% C.I.)	*p*- value	*df*
ACE-III (Total)						
Insufficient	─1.250 (─2.529–0.028)	0.055	1	─0.784 (─1.936- 0.368)	0.182	1
Deficient	─0.313 (─1.400–0.773)	0.572	1	─0.367 (─1.364–0.611)	0.455	1
Attention						
Insufficient	─0.279 (─0.556- ─0.003)	0.047*	1	─0.255 (─0.517–0.007)	0.056	1
Deficient	0.017 (─0.218–0.252)	0.886	1	─0.051 (─0.267–0.173)	0.654	1
Memory						
Insufficient	─0.139 (─0.646–0.367)	0.589	1	─0.003 (─0.487–0.481)	0.99	1
Deficient	0.080 (─0.350–0.510)	0.715	1	0.060 (─0.355–0.476)	0.776	1
Fluency						
Insufficient	─0.494 (─0.893—─0.096)	0.015*	1	─0.322 (─0.694–0.049)	0.089	1
Deficient	─0.464 (─0.803—─0.125)	0.007*	1	─0.336 (─0.655–─0.018)	0.038*	1
Language						
Insufficient	─0.050 (─0.393–0.292)	0.771	1	─0.010 (─0.345–0.324)	0.952	1
Deficient	0.090 (─0.201–0.381)	0.544	1	─0.033 (─0.254–0.320)	0.82	1
Visuospatial						
Insufficient	─0.285 (─0.623–0.052)	0.097	1	─0.193 (─0.509–0.121)	0.228	1
Deficient	─0.036 (─0.323–0.250)	0.803	1	─0.082 (─0.352–0.188)	0.551	1
HMSE						
Insufficient	─0.026 (─0.198–0.146)	0.765	1	─0.028 (─0.200–0.142)	0.742	1
Deficient	0.011 (─0.135–0.159)	0.874	1	─0.029 (─0.177–0.118)	0.694	1

Model 1: Not adjusted for covariates.

Model 2: Adjusted for age, education, gender and dyslipidemia status.

*ACE-III* Addenbrooke’s cognitive examination-III; *HMSE* Hindi mental status examination;

(*) indicates significant at *p* < 0.05.

## Discussion

The results of our study indicate that most of the participants had suboptimal levels of VitD (58% deficient and 23% insufficient). The mean VitD level of the cohort was also below optimal levels (21.54 ± 12.60 ng/ml). These results imply that the urban adult population in southern India had an overall inadequacy of VitD. Our results are in line with the SANSCOG (Srinivaspura Aging, Neurosenescence and COGnition) study, where Sundarakumar et al. reported the burden of VitD inadequacy in a rural population from southern India^35^. Similar findings have been also observed in a previous study from northern India, where high levels of VitD deficiency (91.2%) was reported in healthy adult population above 50 years of age^36^. The prevalence of VitD deficiency ranges between 50 and 94% as reported from several community-based studies on VitD in India^37^. Hospital based studies in India reported a prevalence of VitD deficiency ranging from 37 to 99%^37^. Previous population-based studies provide evidence that the problem of VitD inadequacy in India has existed for nearly two decades^38,39^. The concerning amount of low VitD levels in our cohort also opposes the traditional view that individuals from tropical countries have adequate levels of VitD due to yearlong exposure of these regions to direct sunlight. A recent study from Ecuador have also reported similar findings with high prevalence of VitD deficiency despite being a tropical country^40^. The possible explanation of this could be short duration of exposure to sun due to the generally hot climate of India, limited outdoor activities in the aging population, extensive clothing, air-conditioning in urban households and popularity of sunscreens^41,42^. India has a huge number (39%) of vegetarian population which limits the intake of dietary VitD from animal food sources^41,43^.

Previous studies have suggested a link between growing age and decreasing levels of VitD^36,44^. Adults who lack direct exposure to sunlight are also at risk of developing VitD deficiency which in turn might negatively impact other physiological mechanisms^45^. We observed similar findings in our cohort, where age and levels of VitD had a significant association between them. Apart from sun exposure, diet forms an integral part of maintaining VitD levels in the human body. Seafood, egg yolk, meat, mushrooms and dairy products are rich sources of dietary VitD^1^. Diet patterns did not vary between the individuals in our cohort; thus, no significant effect of diet was seen on VitD status. Females are prone to have VitD deficiency in general^46^. In our cohort we observe a significant sex difference in VitD levels, although more men are found to be deficient than females. Similar findings have been reported by a study from Latin America where the prevalence of VitD deficiency was more in males than females^47^.

The existing body of literature suggests an association of VitD deficiency with an increased risk for several diseases like hypertension, diabetes and dyslipidemia. Adequate levels of VitD often act as a protective factor against development of the above-mentioned diseases which are independent risk factors for cognitive impairment^24,48,49^. The individuals in our cohort did not differ significantly with respect to hypertension and diabetes, although dyslipidemia was found in a large number of individuals with low levels of VitD. Similar findings were reported by a study in Saudi Arabia where an association was found between VitD deficiency and dyslipidemia^50^. Individuals with VitD deficiency in our cohort had abnormal levels of triglycerides, HDL as well as LDL when compared to individuals with normal VitD levels. Our findings resonate with previous studies which reported significant association of VitD deficiency with high LDL and triglyceride levels^51,52^. A meta-analysis by Radkhah et al. found VitD supplementation to help improve lipid profiles, thus strengthening the importance of VitD in maintenance of serum lipid levels^53^. VitD is known to aid lipid metabolism by increasing calcium levels in the blood^48^. Liu et al. suggested that VitD deficiency had a negative impact on cholesterol metabolism which might further give rise to several cardiovascular conditions which are independent risk factors of cognitive decline^54^. Therefore, low levels of VitD are often associated with poor lipid metabolism. Previous studies have suggested dyslipidemia in mid-life to be a risk factor in the development of cognitive impairment and dementia in later life^55,56^. Therefore, it becomes imperative to check for dyslipidemia when an individual is VitD deficient.

Low VitD levels are associated with impaired cognitive functioning in several domains, but whether these associations tantamount to causation is still debatable. This makes it worth exploring the role of VitD on cognitive functioning, especially in the adult population as literature suggests VitD levels to decrease with age^44^. According to the results of the present study, low VitD levels did not affect global cognition as evidenced from non-significant HMSE and ACE-III total scores, but individuals with insufficient levels of VitD showed decreased level of attention. Several previous studies have reported that VitD did not affect global cognition, neither improved cognitive functioning on supplementation^13,40,42^. Deficits in attention is a part of aging and it has been found that attention training interventions improved cognitive functioning in individuals with MCI or mild to moderate dementia^57^. VitD deficiency is a well-known biomarker of Attention-Deficit/ Hyperactivity Disorder (ADHD) in children^58^. In older adults, there is evidence of an association between low VitD levels and the decreased ability to focus attention^59^. Our results are also in line with a study in India by Vedak et al., where a significant association was found between low serum VitD levels and the domain of attention^5^. Therefore, more assessments and further research in this domain is desirable to discern the existence of a cause-effect relationship.

The results of our study also revealed an association between suboptimal levels of VitD and verbal fluency. Verbal fluency is closely associated with semantic processing, language, working memory and executive functioning^60,61^. Previous studies have suggested a correlation between low VitD levels and decline in executive functioning and working memory ^62,63^. So, it may be postulated that the association between low levels of VitD and verbal fluency has an indirect relationship with aspects of executive functioning and memory. Though the literature on direct association between verbal fluency and VitD is scarce, a study in Canada reported similar findings where supratherapeutic levels of VitD supplementation significantly improved scores on verbal fluency tasks^7^. Another study by Nerhus et al. suggested VitD deficiency to be associated with poorer verbal fluency and processing speed^64^.

The primary objective of this study was to understand the patterns of VitD in adult population (middle aged and above) and its impact on cognition. The significant burden of VitD deficiency in India posits the need for strategical community level measures like food fortification and vitamin supplementation. VitD fortification of milk has been proved to be safe and effective in children as reported by prospective trials^65,66^. Besides its impact on cognition (verbal fluency and attention), VitD inadequacy has a significant negative impact on lipid metabolism giving rise to conditions like dyslipidemia which might act as a precursor to several other cardiovascular conditions. The findings further confirm the role of VitD in cognition and health and future research is imperative to ascertain a causational relationship.

Our study is not without limitations. The cross-sectional study design might not be suitable for generalizability of the results. We did not have any means of quantifying endogenous production of VitD in our participants. Also, the study sample is restricted to a particular region; a pan India study sample would provide us with a better understanding of the issue of VitD and cognition. The strength of our study in is its large sample size which provides us with some understanding of VitD patterns and the cognitive implications of the same. These findings pave way for public health measures with a focus on primary prevention.

## Conclusion

This study suggests there exists a significant prevalence of VitD deficiency in urban Indian population and lower levels of serum VitD levels are associated with high prevalence of dyslipidemia. VitD deficiency also negatively impacts verbal fluency and attention. Both verbal fluency and attention involve higher cognitive functions and this result provides us with a scope to further investigate the said domains in relation to VitD. The need for food fortification as well as adequate exposure to sunlight may be necessary in city dwelling adult population to mitigate the cognitive deficits as well as other health issues associated with Vitamin D deficiency.

## Data Availability

The datasets generated and/or analysed during the current study are not publicly available as the study is a longitudinal cohort study and is currently ongoing, the data is still being collected and curated and being monitored by the Institutional Ethics Committee (IEC) and Technical Advisory Committee (TAC). Therefore, it is not made public at this point of time. Data request can be directed to the corresponding author Dr. Thomas Gregor Issac who is the PI of TLSA study and data will be shared if approved by the IEC and TAC.
